# Supplementary material for: Behavioral Transition Path and Pivotal Nodes Regulating Attack in Initial Encounters between Unfamiliar Pigs
Source: Animals (Basel). 2024 Sep 6;14(17):2595. doi: 10.3390/ani14172595 (PMC11394634; doi:10.3390/ani14172595)
Supplement: Supplementary file 1 [file animals-14-02595-s001.zip › animals-3140929-supplementary.pdf]

Here are the highest resolution available of Figures 2 and 3 from the article. However, we need to explain that even in the maximum format, the images are not in high definition. This is because the behavior occurred very suddenly and lasted for an extremely short period, making it challenging to capture. We were able to take these photos by capturing the images quickly once the behavior was observed. This means the photos were taken while we were in movement, which may have resulted in some blurriness.

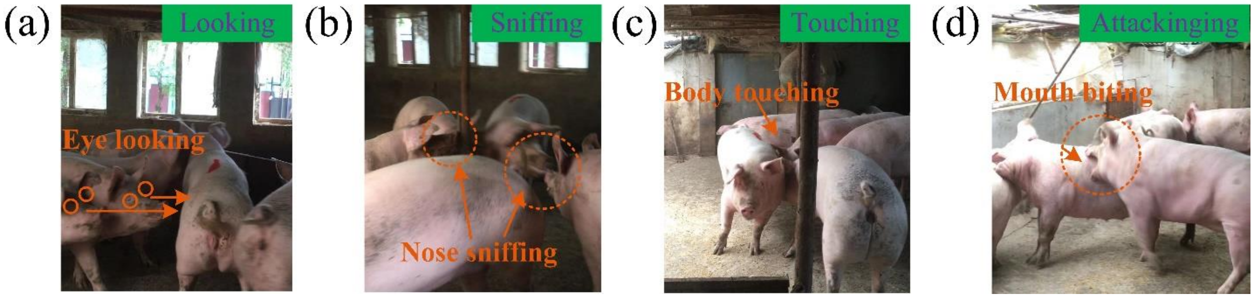

Figure 2. Ethogram of resident pigs encountering the newly added pig. (a) Resident pigs locked their eyes onto the added pig; (b) Resident pig sniffed the odor on the body of the added pig; (c) Resident pigs touched and prodded the added pig's body with their noses; (d) One resident pig opened its mouth to bite the added pig. For high resolution, see Supplementary Materials.

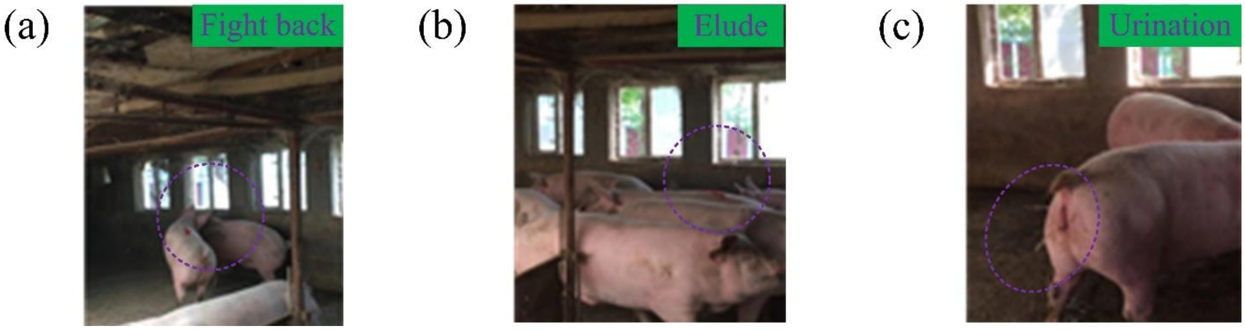

Figure 3. Ethogram of newly added pigs responding to resident pigs. (a) Newly added pigs fought back; (b) Newly added pigs hid within the resident group; and (c) Newly added pigs urinated.
